# Provably Secure Lightweight Mutual Authentication and Key Agreement Scheme for Cloud-Based IoT Environments

**DOI:** 10.3390/s23249766

**Published:** 2023-12-11

**Authors:** Sieun Ju, Yohan Park

**Affiliations:** Department of Computer Engineering, College of Engineering, Keimyung University, Daegu 42601, Republic of Korea; juse0204@gmail.com

**Keywords:** cloud computing, internet of things, authentication, cryptanalysis, real-or-random model, Burrow–Abadi–Needham logic

## Abstract

A paradigm that combines cloud computing and the Internet of Things (IoT) allows for more impressive services to be provided to users while addressing storage and computational resource issues in the IoT environments. This cloud-based IoT environment has been used in various industries, including public services, for quite some time, and has been researched in academia. However, various security issues can arise during the communication between IoT devices and cloud servers, because communication between devices occurs in open channels. Moreover, issues such as theft of a user’s IoT device or extraction of key parameters from the user’s device in a remote location can arise. Researchers interested in these issues have proposed lightweight mutual authentication key agreement protocols that are safe and suitable for IoT environments. Recently, a lightweight authentication scheme between IoT devices and cloud servers has been presented. However, we found out their scheme had various security vulnerabilities, vulnerable to insider, impersonation, verification table leakage, and privileged insider attacks, and did not provide users with untraceability. To address these flaws, we propose a provably secure lightweight authentication scheme. The proposed scheme uses the user’s biometric information and the cloud server’s secret key to prevent the exposure of key parameters. Additionally, it ensures low computational costs for providing users with real-time and fast services using only exclusive OR operations and hash functions in the IoT environments. To analyze the safety of the proposed scheme, we use informal security analysis, Burrows–Abadi–Needham (BAN) logic and a Real-or-Random (RoR) model. The analysis results confirm that our scheme is secure against insider attacks, impersonation attacks, stolen verifier attacks, and so on; furthermore, it provides additional security elements. Simultaneously, it has been verified to possess enhanced communication costs, and total bit size has been shortened to 3776 bits, which is improved by almost 6% compared to Wu et al.’s scheme. Therefore, we demonstrate that the proposed scheme is suitable for cloud-based IoT environments.

## 1. Introduction

**Figure 1:**
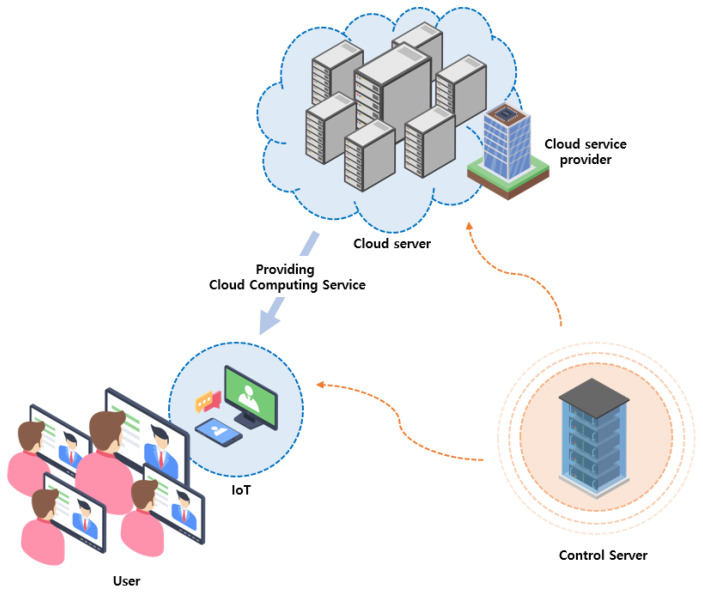
Cloud-based IoT enviroment.

The Internet of Things (IoT) is a network in which Internet-enabled objects interact with each other through the internet [1,2]. IoT objects collect data from their surroundings, provide web services to users, and communicate with each other. Therefore, IoT objects such as smart devices need significant resources to store data collected from sensors and perform real-time computations using limited hardware. Hence, addressing the limitations of storage and computing capacities is crucial for the formation of a network of IoT objects [3,4,5]. However, cloud computing technology refers to the practice of moving computational power and storage space from individual devices to larger shared data centers [6]. Cloud computing allows access to a shared pool of computing resources such as networks, servers, storage, and applications. By using cloud computing, it becomes possible to overcome the limitations inherent in IoT devices [7,8,9,10]. The development and discussion of cloud-based IoT (CloudIoT) have been ongoing since before 2008 and continue to evolve. Figure 1 illustrates the structure of CloudIoT. This structure comprises three entities: user, cloud server, and control server. Users with IoT devices can access the resources provided by the cloud service provider’s server anytime and anywhere through IoT objects. The cloud server collects user’s requests and delivers the right service through IoT. The control server, acting as a trusted entity, generates the necessary parameters for communication between authenticated users and the cloud server through the registration process. Additionally, it monitors the key agreement phase to ensure that users and the cloud server establish the same session key for subsequent communications when needed.

In 2022, Wu et al. [11] proposed a lightweight authentication protocol for IoT-enabled cloud computing environments. The authors argued that their scheme could resist various attacks, such as man-in-the-middle, insider, DDoS, and masquerade attacks, and provides privacy, traceability, and integrity. However, we identified several vulnerabilities in Wu et al.’s scheme, including susceptibility to insider attacks, verification table attacks, user impersonation, and cloud server impersonation. Furthermore, the scheme lacks user untraceability, allowing an attacker to track the same user across different sessions through message eavesdropping alone. To address these vulnerabilities, we propose a provably secure lightweight mutual authentication and key agreement (MAKA) scheme. In our proposed scheme, we protect crucial parameters stored in the user’s IoT smart card using the user’s biometric information to prevent attacks like user impersonation and offline password-guessing. We also enhanced security by adding a secret key to the cloud server, preventing attackers from exploiting leaked database values. Additionally, we reduced the communication and computation overhead by employing only hash functions and exclusive-OR operations.

### 1.1. Research Contributions

We review and conduct a security analysis of Wu et al.’s authentication scheme. We demonstrate that Wu et al.’s scheme is vulnerable to insider attacks, verification table leakage attacks privileged insider attacks, user impersonation, and cloud server impersonation. Additionally, we propose an MAKA for cloud-based IoT environments that leverages biometric information. The proposed scheme is tailored to the IoT environments, using only exclusive OR operations and hash functions to align with a lightweight architecture. Additionally, we use a Real-or-Random (RoR) model and Burrow–Abadi–Needham (BAN) logic to demonstrate formally the security and robustness of the proposed. Moreover, we substantiate the security of our scheme against different attacks, including insider attacks, impersonation attacks, reply and man-in-the-middle (MITM) attacks, privileged insider attacks, ephemeral security leakage, stolen verifier attacks, DoS attacks, and session key disclosure attacks. In addition, we confirmed that our scheme can provide user anonymity, user untraceability, perfect forward secrecy, and mutual authentication. Last, we evaluate the security features, communication costs, and computation costs of the proposed scheme with related schemes, including Wu et al.’s.

### 1.2. Organization

In Section 2, we introduce studies related to cloud-based IoT, IoT, and cloud computing. We present the system model and adversary model used in our proposed scheme in Section 3. Following that, we discuss Wu et al.’s scheme in Section 4. We then delve into the vulnerabilities we identified in Wu et al.’s scheme in Section 5. In Section 6, we introduce our proposed scheme, and in Section 7, we provide security analyses using tools such as BAN logic, and RoR model. Performance analyses, including security features, communication, and computation costs, are presented in Section 8. Finally, in Section 9, we conclude our paper and outline future plans.

## 2. Related Works

When providing services to users over the internet, application security is crucial in gaining user trust. To access various services, including storage services provided by cloud service providers, the environments should be well prepared to handle various attacks and security threats that may exist. Furthermore, in IoT environments, lightweight protocol computations are essential to provide users with a seamless real-time service anytime, anywhere. In the following sections, we will review the authentication protocols in the existing cloud-based IoT environments.

In 2019, Schouqi et al. [12] introduced an authentication protocol for IoT built on Nikooghadam et al.’s [13] protocol. The protocol of Nikooghadam et al. was developed as a responses to issues with the authentication protocol proposed by Kumari et al. [14]. However, Nikooghadam et al.’s scheme has already been analyzed by researchers in the field, including Limbasiya et al., Chandrakar-Om, and Sharma-Kalra [15,16,17]. These researchers raised concerns about its security, highlighting vulnerabilities to various attacks such as password-guessing, insiders, and modification attacks. They also indicated that the protocol lacked forward secrecy and did not provide session key verification and a biometric update phase. The author of the new scheme reviewed the security issues known in Nikooghadam et al.’s protocol and proposed enhancements based on these findings.

Prosanta and Biplab (Prosanta-Biplab) [18] proposed lightweight two-factor authentication scheme for IoT devices in 2019. They argued that two-factor authentication schemes that use a passwords and smartcards, often vulnerable to physical attacks. To overcome these security issues they suggested physically uncloneable functions(PUF) as an authentication factor for IoT devices. However, in 2020, Siddiqui et al. [19] demonstrated that the scheme is vulnerable to man-in-the-middle, impersonations, session- hijacking and conventional and differential template attacks.

In 2019, Zhou et al. [20] presented a lightweight two-factor authentication scheme for IoT devices available in the cloud environments. In the same year, Rafael et al. [21] indicated that Zhou et al.’s scheme has several security issues. Rafael et al. demonstrated that Zhou et al.’s scheme failed to provide mutual authentication, was unsuccessful in protecting the secret key, and was vulnerable to various attacks, including insider attacks and man-in-the-middle attacks.

In 2020, Alzahrani et al. [22] presented an authentication protocol for IoT environments based on self-certified public keys and elliptic curve cryptography (ECC). Alzahrani conducted research on protocols proposed by Islam-Biswas [23] and Mandal et al. [24], highlighting their failure to ensure user anonymity and vulnerability to impersonation attacks. Therefore, the author developed a protocol that guarantees anonymity among connected devices and addresses security vulnerabilities. However, this scheme does not guarantee security against physical attacks.

Chen et al. [25] proposed a lightweight user authentication and key-agreement scheme for IoT. Chen et al. utilized XOR operations, hash functions, and elliptical multiplication. Lee et al. [26] indicated that Chen et al.’s scheme did not provide a steal-resistant smartcard offline password, offline identity guessing and reply attack. Subsequently, in 2020, Ye et al. [27] proposed an authentication and key agreement scheme for IoT-based cloud computing environments by advancing the protocol developed by He et al. [28]. Ye et al. addressed various security issues in the scheme proposed by He et al, such as failure to resist insider attacks, offline password-guessing, user impersonation, and potential DoS attacks.

Table 1, summarizes cryptographic technologies and limitations of various authentication schemes related to IoT, cloud-based IoT, and cloud computing environments. Related papers propose various protocols to provide users with secure and fast services in the CloudIoT environment. However, there are still vulnerabilities and challenges in fully supporting security features, as some attacks persist. Additionally, methods using symmetric keys like ECC may incur higher computation costs in IoT environments. Therefore, our goal is to design a lightweight protocol tailored for IoT environments using XOR and to achieve higher security in our scheme.

## 3. Preliminaries

### 3.1. System Model

**Figure 2:**
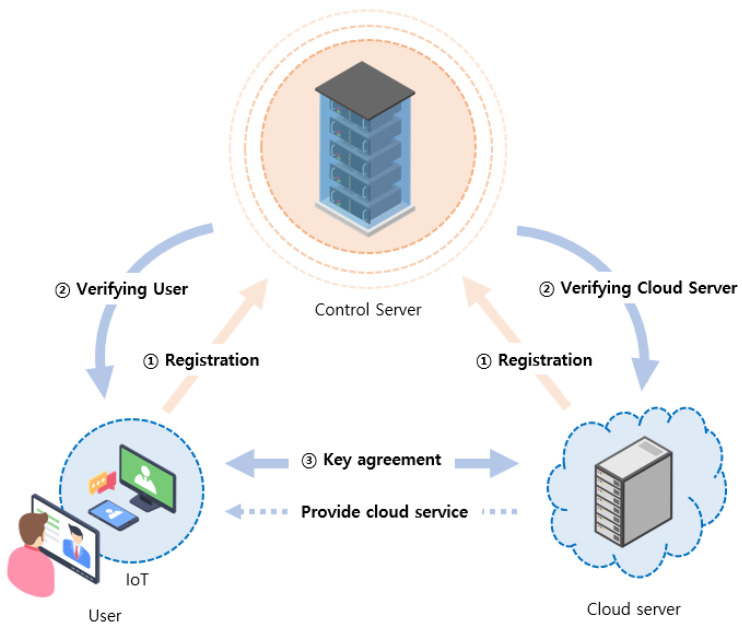
System model.

As shown in Figure 2, an IoT-enable cloud computing environment includes three entities: user, cloud server, and control server. Users can also use cloud computing provided by a cloud server, using IoT-enabled devices. Therefore, the user and cloud server should register and authenticate it through the control server. Finally, the user and cloud server share a session key for communication. The details are as follows

- User ($U_{i}$): User uses IoT devices with cloud services. Communicates with the cloud servers, then the user should register with the control server. The user can use smart cards and biometric technology to store sensitive information or the user’s identity and password. We assumed that the user is an untrusted entity, implying that the user can execute unauthorized or malicious attacks.
- Cloud server ($S_{j}$): A cloud server provides cloud services to users using IoT devices. To achieve this, the cloud server should be registered with the control server. As a semi-trusted entity, the cloud server can misbehave; however, it cannot directly collude or participate.
- Control server (CS): This manages the registration of the user and cloud server, and helps generate the session key for authentication and subsequent communication. As a semi-trusted entity, the control server can misbehave; however, it cannot directly collude or participate.

### 3.2. Adversary Model

We employ the widely used “Dolev–Yao (DY) model” [32] to define the capabilities of the adversary. The details are as follows:

- Within the DY model, entities in the IoT environments are considered trustworthy, and the communication channel is also considered insecure. Consequently, the adversary can engage in various actions through the insecure channel, including resending, eavesdropping, blocking, and deleting any messages transmitted.
- The adversary can extract sensitive information through power analysis attacks from stolen user smart cards. Additionally, because the control and cloud servers are semi-trusted entities, the adversary can also extract information from their databases.

Furthermore, the “Canetti–Krawczyk (CK) model” [33] assumes that stronger adversaries can also be adapted to our protocol. The adversaries in the CK model can obtain and use ephemeral values or long-term values, and using those, ephemeral leakage attacks can be performed.

## 4. Revisit of Wu et al.’s Scheme

### 4.1. Registration Phase

Before generating a session key for communication, the user and cloud server must go through the registration process via a secure channel. The detailed process is as follows.

#### 4.1.1. User Registration Phase

**Step 1:**  The $U_{i}$ enters $ID_{i}$, $PW_{i}$ and imprints $B_{i}$ on the device. Then, calculates $Gen\left(B_{i}\right)=\sigma_{i},\tau_{i}$, $HPW_{i}=h(PW_{i}\left|\right|\sigma_{i})$ and sends $ID_{i},HPW_{i}$ to CS as a registration request message through a secure channel.
**Step 2:**  CS checks if $U_{i}$’s identity is new, and generates a random number $n_{i}$ to calculate $TID_{i}=h\left(ID_{i}\right)$, $A_{1}=h(ID_{CS}||HPW_{i})⊕\left(n_{i}⊕x\right)$. Then, stores $\left\{TID_{i},HPW_{i}\right\}$ in its database, and stores $\left\{A_{1},ID_{CS}\right\}$ to smart card SC. After that, sends SC to $U_{i}$ through a secure channel.
**Step 3:**  $U_{i}$ computes $A_{2}=h(ID_{i}||HPW_{i})$ and store $\left\{A_{1},A_{2},ID_{CS},Gen\left(\cdot \right),Rep\left(\cdot \right),\tau_{i}\right\}$ in SC.

#### 4.1.2. Cloud Server Registration Phase

**Step 1:**  $S_{j}$ selects $SID_{j}$ and random number $n_{j}$ and sends $\left\{SID_{j},n_{j}\right\}$ as a request message to CS through a secure channel.
**Step 2:**  CS checks if $S_{j}$’s identity is new, and chooses $S_{j}$’s pseudo identity $QID_{j}$, computes $A_{3}=h(SID_{j}||x⊕n_{j})$, then stores $\left\{QID_{j},n_{j}\right\}$ in its database. Next, CS sends $QID_{j},n_{j}$ to $S_{j}$ through secure channel.
**Step 3:**  $S_{j}$ computes $A_{3}^{*}=A_{3}⊕SID_{j}$, and stores $\left\{A_{3}^{*},QID_{j}\right\}$.

### 4.2. Login and Authentication Phase

**Figure 3:**
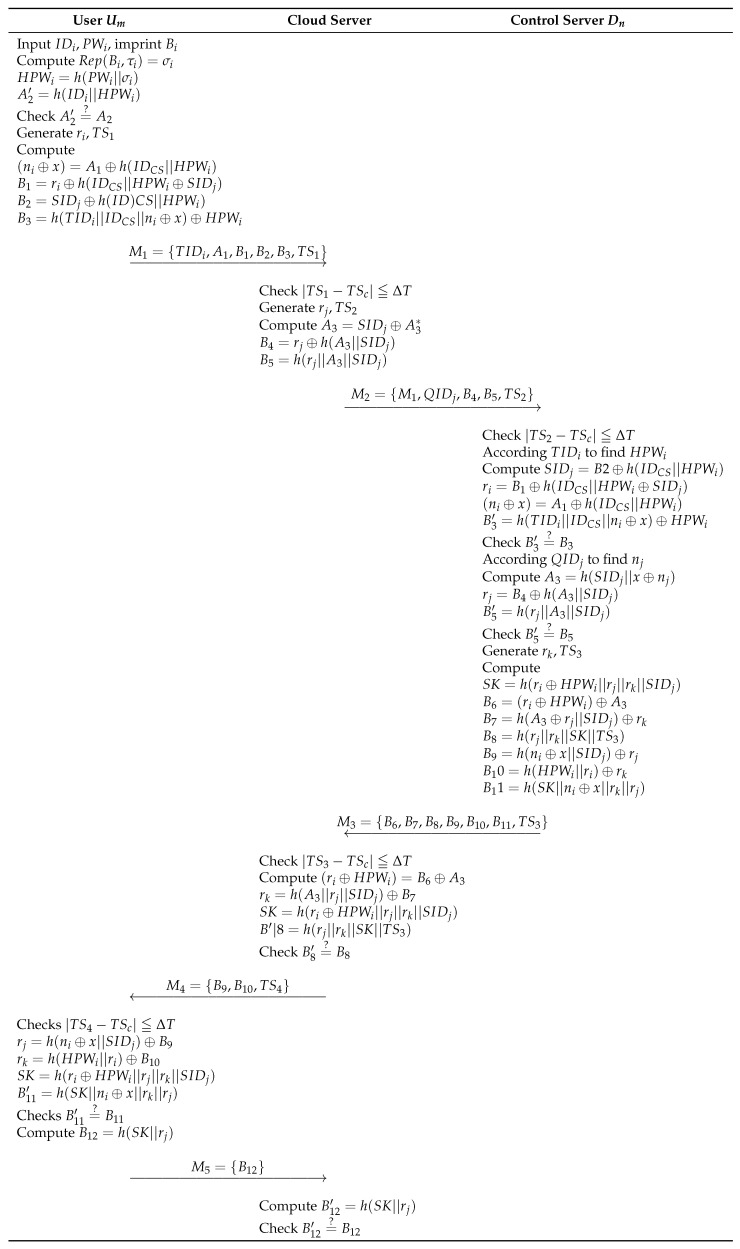
AKA phase of Wu et al.’s scheme.

In this phase, the control server first verifies the identities of the user and the cloud server. If both are confirmed, a shared session key for subsequent communication is generated. The detailed process is as follows and illustrated in Figure 3.

**Step 1:**  $U_{i}$ enters $ID_{i}$, $PW_{i}$ and imprints $B_{i}$, and calculates $Rep\left(B_{i},\tau_{i}\right)=\sigma_{i}$, $HPW_{i}=h(PW_{i}\left|\right|\sigma_{i})$, $A^{\prime }2=h(ID_{i}||HPW_{i})$. Then, by confirming $A_{2}^{\prime }\overset{?}{=}A2$, $U_{i}$ can be verified as a legitimate user. If this is valid, $U_{i}$ selects a random number $r_{i}$ and timestamp $TS_{1}$, then calculates $\left(n_{i}⊕x\right)=A_{1}⊕h(ID_{CS}||HPW_{i})$, $B_{1}=r_{i}⊕h(ID_{CS}||HPW_{i}⊕SID_{j})$, $B_{2}=SID_{j}⊕h(ID_{CS}||HPW_{i})$, and $B_{3}=h(TID_{i}||ID_{CS}\left|\right|n_{i}⊕x)⊕HPW_{i}$. Finally, generates a message $M_{1}=\left\{TID_{i},A_{1},B_{1},B_{2},B_{3},TS_{1}\right\}$ and sends it to $S_{j}$ via open channel.
**Step 2:**  Upon receiving $U_{i}$’s message, CS confirms timestamp $|TS_{1}-TS_{c}|\;≦\;\Delta T$. If the timestamp is valid, $S_{j}$ chooses a random value $r_{j}$ and timestamp $TS_{2}$. $S_{j}$ computes $A_{3}=SID_{j}⊕A_{3}^{*}$, $B_{4}=r_{j}⊕h(A_{3}||SID_{j})$, and $B_{5}=h(r_{j}\left|\right|A_{3}||SID_{j})$. Finally, message $M_{2}=\left\{M_{1},QID_{j},B_{4},B_{5},TS_{2}\right\}$ is sent through an open channel.
**Step 3:**  After receiving the $M_{2}$, $S_{j}$ confirms timestamp $|TS_{2}-TS_{c}|\;≦\;\Delta T$. If the timestamp is successfully verified, CS uses $TID_{i}$ to find $HPW_{i}$ and performs the following computations: $SID_{j}=B_{2}⊕h(ID_{CS}||HPW_{i})$, $r_{i}=B_{1}⊕h(ID_{CS}||HPW_{i}⊕SID_{j})$, and $B_{3}^{\prime }=h(TID_{i}||ID_{CS}\left|\right|n_{i}⊕x)⊕HPW_{i}$. And by checking $B_{3}^{\prime }\overset{?}{=}B_{3}$, the CS confirms whether $U_{i}$ is the legitimate user. Next, CS utilizes the value of $QID_{j}$ to find $n_{j}$ and then performs the following computations: $A_{3}=h(SID_{j}||x⊕n_{j})$, $r_{j}=B_{4}⊕h(A_{3}||SID_{j})$, and $B_{5}^{\prime }=h(r_{j}\left|\right|A_{3}||SID_{j})$. After checking $B_{5}^{\prime }\overset{?}{=}B_{5}$ is valid, CS then selects $r_{k}$, $TS_{3}$, computes $SK=h(r_{i}⊕HPW_{i}||r_{j}||r_{k}||SID_{j})$, $B_{6}=\left(r_{i}⊕HPW_{i}\right)⊕A_{3}$, $B_{7}=h(A_{3}\left|\right|r_{j}||SID_{j})⊕r_{k}$, $B_{8}=h(r_{j}\left|\right|r_{k}||SK||TS_{3})$, $\left(n_{i}⊕x\right)=A_{1}⊕h(ID_{CS}||HPW_{i})$, $B_{9}=h(n_{i}⊕x||SID_{j})⊕r_{j}$, and $B_{10}=h(HPW_{i}\left|\right|r_{i})⊕r_{k}$, $B_{11}=h(SK||n_{i}⊕x||r_{k}\left|\right|r_{j})$. At last, CS generates message $M_{3}=\left\{B_{6},B_{7},B_{8},B_{9},B_{10},B_{11},TS_{3}\right\}$ and sends to $S_{j}$ through an open channel.
**Step 4:**  Upon receiving $M_{3}$, $S_{j}$ checks timestamp $|TS_{3}-TS_{c}|\;≦\;\Delta T$. If the timestamp is valid, $S_{j}$ calculates following computations: $\left(r_{i}⊕HPW_{i}\right)=B_{6}⊕A_{3}$, $SK=h(r_{i}⊕HPW_{i}||r_{j}||r_{k}||SID_{j})$, and $B_{8}^{\prime }=h(r_{j}\left|\right|r_{k}||SK||TS_{3})$, and confirms $B_{8}^{\prime }\overset{?}{=}B_{8}$. If it confirms, $S_{j}$ generates message $M_{4}=\left\{B_{9},B_{10},TS_{4}\right\}$ to $U_{i}$ via open channel.
**Step 5:**  $U_{i}$ verifies timestamp $|TS_{4}-TS_{c}|\;≦\;\Delta T$. If the timestamp is valid, $U_{i}$ calculates $r_{j}=h(n_{i}⊕x||SID_{j})⊕B_{9}$, $r_{k}=h(HPW_{i}\left|\right|r_{i})⊕B_{10}$, $SK=h(r_{i}⊕HPW_{i}||r_{j}||r_{k}||SID_{j})$, and $B_{11}^{\prime }=h(SK||n_{i}⊕x||r_{k}\left|\right|r_{j})$ and calculates $B_{11}^{\prime }\overset{?}{=}B_{11}$. If it confirms, $U_{i}$ computes $B_{12}=h(SK||r_{j})$ and generates $M_{5}=\left\{B_{12}\right\}$ and sends to $S_{j}$.
**Step 6:**  $S_{j}$ calculates the equation $B_{12}^{\prime }=h(SK||r_{j})$ and then checks $B_{12}^{\prime }\overset{?}{=}B_{12}$. If they match, $S_{j}$ stores SK for future communication.

## 5. Cryptanalysis of Wu et al.’s Scheme

Following the description of Section 3, adversary A can obtain important values from the user’s smart card by using a power analysis attack. Furthermore, A can extract parameters from the cloud server and control server itself, because they are considered semi-trusted. With this information, various security attacks, including insider attack, verification table leakage attack, privileged insider attack, user impersonation, and cloud server impersonation, can be executed by A. Details are described below.

### 5.1. Insider Attack

An adversary *A*, who has undergone the registration process as a legitimate user, can obtain session keys from another user $U_{i}$’s sessions or impersonate $U_{i}$. The detailed process is as follows:

**Step 1:**  After completing the registration process, *A* obtains $B_{6}$ of $M_{3}$ during their AKA process. Subsequently, *A* calculates $A_{3}$ of $S_{j}$ using their own $HPW_{a}$ and $r_{a}$.
**Step 2:**  In another user $U_{i}$’s session, *A* obtains message $M_{2}$ and uses $B_{4}$ and the previously acquired $A_{3}$ to deduce $r_{j}$.
**Step 3:**  From B${}_{6}$ of $M_{3}$, *A* calculates user $U_{i}$’s $r_{i}$ and $HPW_{i}$, and from $B_{7}$, *A* calculates $r_{k}$.
**Step 4:**  Using the computed values, A can generate the session key $SK=h(r_{i}⊕HPW_{i}||r_{j}$$\left|\right|r_{k}||SID_{j})$ for another user $U_{i}$ and potentially disclose or exploit it.

Therefore, Wu et al.’s scheme cannot resist insider attacks.

### 5.2. Verification Table Leakage Attack

If A extracts verification table of cloud server, A can disclose session key. The following procedures are below:

**Step 1:**  A extracts the verification table to take $\left\{A_{3}^{*},QID_{j}\right\}$ from $S_{j}$. And also intercept message $M_{2}=\left\{M_{1},QID,B_{4},B_{5},TS_{2}\right\}$ transmitted in public channel.
**Step 2:**  A calculates $A_{3}=SID_{j}⊕A_{3}^{*}$, and $r_{j}=B_{4}⊕h(A_{3}||SID_{j})$ to extract $A_{3}$ and $r_{j}$.
**Step 3:**  A takes message $M_{3}=\left\{B_{6},B_{7},B_{8},B_{9},B_{10},B_{11},TS_{3}\right\}$.
**Step 4:**  A computes $\left(r_{i}⊕HPW_{i}\right)=B6⊕A_{3}$, and $r_{k}=h(A_{3}\left|\right|r_{j}||SID_{j})⊕B_{7}$. In addition by calculating $SK=h(r_{i}⊕HPW_{i}||r_{j}||r_{k}||SID_{j})$, A can generate a session key to disclose or exploit it.

Therefore, Wu et al.’s scheme cannot resist verification table leakage attacks.

### 5.3. Privileged Insider Attack

A privileged insider can take important information like $\left\{ID_{j},HPW_{j}\right\}$ from the registration message and values stored in the user’s smart card such as $\left\{A_{1},A_{2},ID_{cs},Gen\left(\right),Rep\left(\right),\right\}$. Through support from this privileged insider, a malicious A can generate a session key through the following:

**Step 1:**  A computes $SID_{j}=B_{2}⊕h(ID_{CS}||HPW_{i})$, and $r_{i}=B_{1}⊕h(ID_{CS}||HPW_{i}⊕SID_{j})$, $\left(n_{i}⊕x\right)=A_{1}⊕h(ID_{CS}||HPW_{i})$; therefore, A can extract parameters $SID_{j}$, $r_{i}$, and $\left(n_{i}⊕x\right)$.
**Step 2:**  A intercepts message $M_{4}=\left\{B_{9},B_{10},TS_{4}\right\}$.
**Step 3:**  A calculates $r_{j}=h(n_{i}⊕x||SID_{j})⊕B_{9}$, and $r_{k}=h(HPW_{i}\left|\right|r_{i})⊕B_{10}$. Hence, A can compute session key $SK=h(r_{i}⊕HPW_{i}||r_{j}||r_{k}||SID_{j})$ and disclose it.

Thus, Wu et al.’s scheme is insecure against privileged insider attacks.

### 5.4. Impersonation

When A obtains the table information $\left\{k_{n},SID_{n}\right\}$ of the control center, A can calculate $SK_{nm}=h(SID_{m}||SID_{n}||SID_{c}\left|\right|A_{8})$.

(1) User impersonation: If the privileged insider described in Section 5.3 generates random number $r_{i}$ and time stamp $TS_{1}$, A can forge message $M_{1}=\left\{TID_{i},A_{1},B_{1},B_{2},B_{3},TS_{1}\right\}$. In addition, by A to take message $M_{4}=\left\{B_{9},B_{10},TS_{4}\right\}$ from an unsecured public channel, A can generate session key and rj. Thus, A can send message $M_{5}=\left\{B12\right\}$ impersonates user.
(2) Cloud server impersonation: According to the previous verification table attack in Section 5.2, A generates random number $r_{j}$ and time stamp $TS_{2}$, and A can send $M_{2}=\left\{M_{1},QID_{j},B_{4},B_{5},TS_{2}\right\}$. Second, A can generate $M_{4}=\left\{B_{9},B_{10},TS_{4}\right\}$ after intercept message $M_{3}=\left\{B_{6},B_{7},B_{8},B_{9},B_{10},B_{11},TS_{3}\right\}$. Hence A can impersonate cloud server.

Therefore, Wu et al.’s scheme cannot resist user and cloud impersonation attack.

### 5.5. Lack of Untraceability

If an attacker *A* continues to eavesdrop on $M_{1}=\left\{TID_{i},A_{1},B_{1},B_{2},B_{3},TS_{1}\right\}$ and compares the value of $TID_{i}$ contained in $M_{1}$, *A* can track the user $U_{i}$. The reason is that the pseudo identity of $U_{i}$, $TID_{i}$, is a fixed value, and an attacker can easily obtain it through eavesdropping on the message. Indeed, by verifying whether the value of $TID_{i}$ matches the values from previous or subsequent communications, *A* can detect the user. In conclusion, Wu et al.’s scheme lacks anonymity and untraceability.

### 5.6. Impossibility of Offline Password Update

In the user registration phase in Wu et al.’s scheme, the value of $HPW_{i}$ is created by concatenating the user’s password $PW_{i}$ with their biometric information $\sigma_{i}$. Additionally, this $HPW_{i}$ is transmitted to the control server CS and undergoes the operation $A_{1}=h(ID_{CS}||HPW_{i})⊕\left(n_{i}⊕x\right)$, and stored in the CS’s database as $A_{1}$. However, this design leads to a problem where users must communicate with the CS to update the $A_{1}$ value stored in the CS if they wish to change their password, because CS cannot create the $HPW_{i}$ on its own. Consequently, Wu et al.’s scheme does not support offline password updates.

## 6. Proposed Protocol

### 6.1. Registration Phase

**Figure 4:**
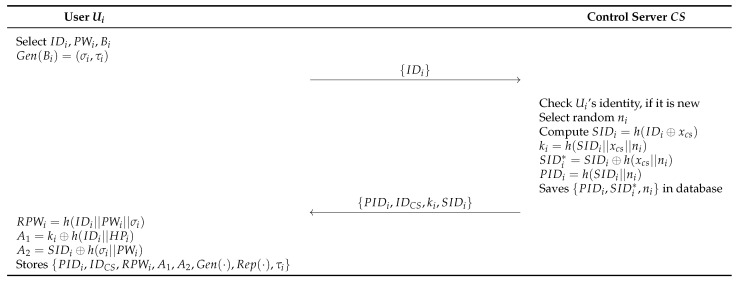
User registration phase of proposed scheme.

Before generating a session key for communication, the user and the cloud server must go through the registration process with the control server via a secure channel. In this phase, users register the information, such as identity, password, and biometrics, with the control server. The detailed process is as follows and illustrated in Figure 4.

#### 6.1.1. User Registration Phase

**Step 1:**  The $U_{i}$ enters $ID_{i}$, $PW_{i}$ and imprints $B_{i}$ on the device. Then, calculates $Gen\left(B_{i}\right)=\sigma_{i}$ and sends $ID_{i}$ to CS as a registration request message through a secure channel.
**Step 2:**  CS checks if $U_{i}$’s identity is new, and generates a random number $n_{i}$ to calculate $SID_{i}=h(ID_{i}⊕x_{cs}$, $k_{i}=h(SID_{i}\left|\right|x_{cs}\left|\right|n_{i})$. $SID_{i}^{*}=SID_{i}⊕h(x_{cs}\left|\right|n_{i})$ and $PID_{i}=h(SID_{i}\left|\right|n_{i})$. Then, stores $\left\{PID_{i},SID_{i}^{*},n_{i}\right\}$ in its database, and sends $\left\{PID_{i},ID_{cs},k_{i},SID_{i}\right\}$ to $U_{i}$ through secure channel.
**Step 3:**  $U_{i}$ computes $RPW_{i}=h(ID_{i}||PW_{i}\left|\right|\sigma_{i})$, $A_{1}=k_{i}⊕h(ID_{i}||HP_{i})$, and $A_{2}=SID_{i}⊕h(\sigma_{i}||PW_{i})$. Then, store $\left\{PID_{i},ID_{CS},RPW_{i},A_{1},A_{2},Gen\left(\cdot \right),Rep\left(\cdot \right),\tau_{i}\right\}$ in SC.

#### 6.1.2. Cloud Server Registration Phase

**Figure 5:**
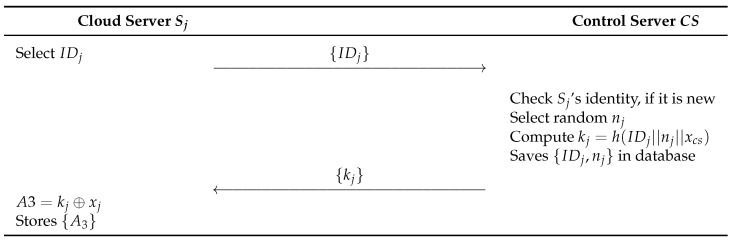
Cloud server registration phase of proposed scheme.

In this phase, cloud servers register the information with the control server. The detailed process is as follows and illustrated in Figure 5.

**Step 1:**  $S_{j}$ selects $SID_{j}$ and sends $\left\{ID_{j}\right\}$ as a request message to CS through a secure channel.
**Step 2:**  CS checks if $S_{j}$’s identity is new, and chooses random number $n_{j}$, computes $k_{j}=h(ID_{j}\left|\right|n_{j}\left|\right|x_{cs})$. Then, stores $\left\{ID_{j},n_{j}\right\}$ in its database. Next, CS sends $k_{j}$ to $S_{j}$ through secure channel.
**Step 3:**  $S_{j}$ computes $A_{3}=k_{j}⊕x_{j}$, and stores $\left\{A_{3}\right\}$.

### 6.2. Login and Authentication Phase

**Figure 6:**
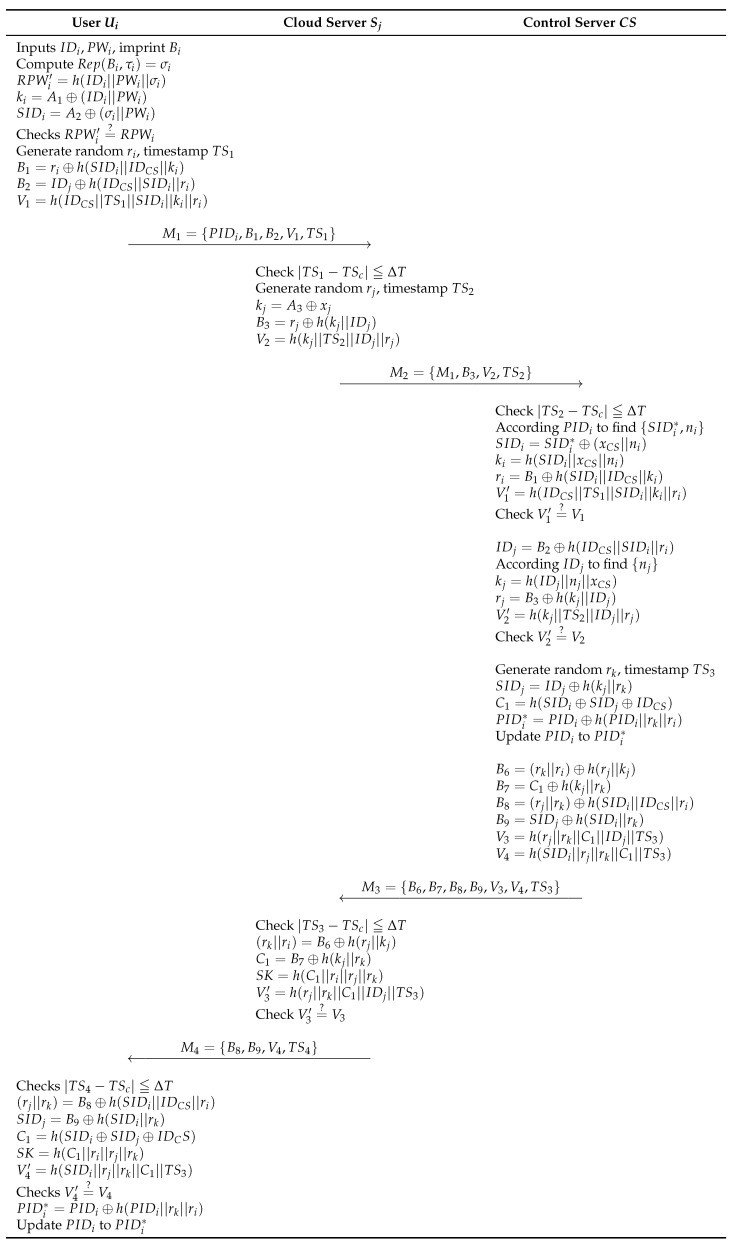
AKA phase of proposed scheme.

In this phase, the control server first verifies the identities of the user and the cloud server. If both are confirmed, a shared session key for subsequent communication is generated. The detailed process is as follows and illustrated in Figure 6.

**Step 1:**  $U_{i}$ enters $ID_{i}$, $PW_{i}$, imprints $B_{i}$, and calculates $Rep\left(B_{i},\tau_{i}\right)=\sigma_{i}$, $RPW_{i}^{\prime }=h(ID-I||PW_{i}\left|\right|\sigma_{i})$, $k_{i}=A_{1}⊕(ID_{i}||PW_{i})$ and $SID_{i}=A_{q}⊕(\sigma_{i}||PW_{i})$. Then, by confirming $RPW_{i}^{\prime }\overset{?}{=}RPW_{i}$, $U_{i}$ can be verified as a legitimate user. If this is valid, $U_{i}$ selects a random number $r_{i}$ and timestamp $TS_{1}$ then calculates $B_{1}=r_{i}⊕h(SID_{i}||ID_{CS}\left|\right|k_{i})$, $B_{2}=ID_{j}⊕h(ID_{CS}||SID_{i}\left|\right|r_{i})$ and $V_{1}=h(ID_{CS}||TS_{1}||SID_{i}\left|\right|k_{i}\left|\right|r_{i})$. Finally, it generates a message $M_{1}=\left\{PID_{i},B_{1},B_{2},V_{1},TS_{1}\right\}$ and sends it to $S_{j}$ via open channel.
**Step 2:**  Upon receiving $U_{i}$’s message, CS confirms timestamp $|TS_{1}-TS_{c}|\;≦\;\Delta T$. If thetimestamp is valid, $S_{j}$ chooses a random value $r_{j}$ and timestamp $TS_{2}$. $S_{j}$ computes $k_{j}=A_{3}⊕x_{j}$, $B_{3}=r_{j}⊕h(k_{j}||ID_{j})$, and $V_{2}=h(k_{j}||TS_{2}||ID_{j}\left|\right|r_{j})$. Finally, message $M_{2}=\left\{M_{1},B_{3},V_{2},TS_{2}\right\}$ is sent through an open channel.
**Step 3:**  After receiving the $M_{2}$, $S_{j}$ confirms timestamp $|TS_{2}-TS_{c}|\;≦\;\Delta T$. If the timestamp is successfully verified, CS uses $PID_{i}$ to find $\left\{SID_{i}^{*},n_{i}\right\}$ and performs the following computations: $SID_{i}=SID_{i}^{*}⊕(x_{CS}\left|\right|n_{i})$, $k_{i}=h(SID_{i}\left|\right|x_{CS}\left|\right|n_{i})$, $r_{i}=B_{1}⊕h(SID_{i}||ID_{CS}\left|\right|k_{i})$ and $V_{1}^{\prime }=h(ID_{CS}||TS_{1}||SID_{i}\left|\right|k_{i}\left|\right|r_{i})$. And by checking $V_{1}^{\prime }\overset{?}{=}V_{1}$, the CS confirms whether $U_{i}$ is the legitimate user.
**Step 4:**  Next, CS calculates $ID_{j}=B_{2}⊕h(ID_{CS}||SID_{i}\left|\right|r_{i})$ and utilizes the value of $ID_{j}$ to find $n_{j}$. Then, it performs the following computations: $k_{j}=h(ID_{j}\left|\right|n_{j}\left|\right|x_{CS})$, $r_{j}=B_{3}⊕h(k_{j}||ID_{j})$, and $V_{2}^{\prime }=h(k_{j}||TS_{2}||ID_{j}\left|\right|r_{j})$. Subsequently, it checks if $V_{2}^{\prime }\overset{?}{=}V_{2}$ is valid.
**Step 5:**  CS then selects $r_{k}$, $TS_{3}$, computes $SID_{j}=ID_{j}⊕h(k_{j}\left|\right|r_{k})$, $C_{1}=h\left(SID_{i}⊕SID_{j}⊕ID_{CS}\right)$, $PID_{i}^{*}=PID_{i}⊕h(PID_{i}\left|\right|r_{k}\left|\right|r_{i})$. Next, it updates the old $PID_{i}$ to $PID_{i}^{*}$.
**Step 6:**  $B_{6}=(r_{k}\left|\right|r_{i})⊕h(r_{j}\left|\right|k_{j})$, $B_{7}=C_{1}⊕h(k_{j}\left|\right|r_{k})$, $B_{8}=(r_{j}\left|\right|r_{k})⊕h(SID_{i}||ID_{CS}\left|\right|r_{i})$, $B_{9}=SID_{j}⊕h(SID_{i}\left|\right|r_{k})$, $V_{3}=h(r_{j}\left|\right|r_{k}\left|\right|C_{1}||ID_{j}||TS_{3})$ and $V_{4}=h(SID_{i}\left|\right|r_{j}\left|\right|r_{k}\left|\right|C_{1}\left|\right|$$TS_{3})$. At last, CS generates message $M_{3}=\left\{B_{6},B_{7},B_{8},B_{9},V_{3},V_{4},TS_{3}\right\}$ and sends to $S_{j}$ through an open channel.
**Step 7:**  Upon receiving $M_{3}$, $S_{j}$ checks timestamp $|TS_{3}-TS_{c}|\;≦\;\Delta T$. If the timestamp is valid, $S_{j}$ calculates following computations: $(r_{k}\left|\right|r_{i})=B6oplush(r_{j}\left|\right|k_{j})$, $C_{1}=B_{7}⊕h(k_{j}\left|\right|r_{k})$, $SK=h(C_{1}||r_{i}||r_{j}||r_{k})$, and $V_{3}^{\prime }=h(r_{j}\left|\right|r_{k}\left|\right|C_{1}||ID_{j}||TS_{3})$. Subsequently, it confirms $V_{3}^{\prime }\overset{?}{=}V_{3}$. If it confirms, $S_{j}$ generates message $M_{4}=\left\{B_{8},B_{9},V_{4},TS_{4}\right\}$ to $U_{i}$ via an open channel.
**Step 8:**  $U_{i}$ verifies timestamp $|TS_{4}-TS_{c}|\;≦\;\Delta T$. If the timestamp is valid, $U_{i}$ calculates $(r_{j}\left|\right|r_{k})=B_{8}⊕h(SID_{i}||ID_{CS}\left|\right|r_{i})$, $SID_{j}=B_{9}⊕h(SID_{i}\left|\right|r_{k})$, $C_{1}=h\left(SID_{i}⊕SID_{j}⊕ID_{CS}\right)$, $SK=h(C_{1}||r_{i}||r_{j}||r_{k})$, and calculates $V_{4}^{\prime }=h(SID_{i}\left|\right|r_{j}\left|\right|r_{k}\left|\right|C_{1}||TS_{3})$ to check $B_{11}^{\prime }\overset{?}{=}B_{11}$. If it confirms, $U_{i}$ computes $PID_{i}^{*}=PID_{i}⊕h(PID_{i}\left|\right|r_{k}\left|\right|r_{i})$ and update old $PID_{i}$ to $PID_{i}^{*}$.

### 6.3. Offline Password and Biometric Template Update

**Figure 7:**
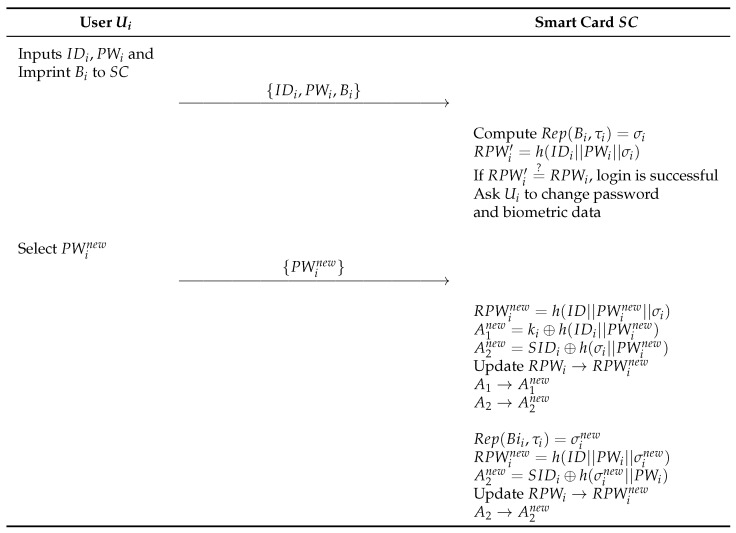
Offline password and biometric template update of proposed scheme.

In this phase, an authenticated user *U* can locally change their password and biometrics without a connection to CS. *U* must perform the login process on the IoT device before updating data offline. A logged-in user can update their password or biometric template. The detailed process is as follows and illustrated in Figure 7.

**Step 1:**  $U_{i}$ enters $ID_{i}$, $PW_{i}$ and imprint $B_{i}$ on the device. Compute $Rep\left(Bio_{i},\tau_{i}\right)=\sigma_{i}$ and check $RPW_{i}^{\prime }=h(ID_{i}||PW_{i}\left|\right|\sigma_{i})$ for login phase and confirm user.
**Step 2:**  Then, ask $U_{i}$ to change password and biometric data. $U_{i}$ select new password $PW_{i}^{new}$, and compute $RPW_{i}^{new}=h(ID_{i}||PW_{i}^{new}\left|\right|\sigma_{i})$, $A_{1}^{new}=k_{i}⊕h(ID_{i}||PW_{i}^{new})$ and $A_{2}^{new}=SID_{i}⊕h({⊕}_{i}||PW_{i}^{new})$. Subsequently, update $RPW_{i}$, $A_{1}$, and $A_{2}$ with new data to change the password.
**Step 3:**  Compute $Rep\left(Bi_{i},\tau_{i}\right)=\sigma_{i}^{new}$, $RPW_{i}^{new}=h(ID||PW_{i}\left|\right|\sigma_{i}^{new})$ and $A_{2}^{new}=SID_{i}⊕h(\sigma_{i}^{new}||PW_{i})$. Subsequently, update $RPW_{i}^{new}$ and $A_{2}^{new}$ with new data to change the biometric template.

## 7. Security Analysis

### 7.1. ROR Model

In this section, we conduct an analysis of session key security using the ROR model [34]. To apply the proposed protocol to the ROR model, we first define participants, especially $U_{US}^{i_{1}}$, $U_{SJ}^{i_{2}}$, and $U_{CS}^{i_{3}}$ as user, cloud server, and control server, respectively. Note that $i_{k}$(k=1,2,3) is an instance for each participant. In ROR model, the adversary can eavesdrop, delete, intercept, and send messages through the public channel. Moreover, the adversary can extract secret parameters from the user $U_{US}^{i_{1}}$. These actions of the adversary can be defined as queries in the ROR model.

- $EX(U_{US}^{i_{1}},U_{SJ}^{i_{2}},U_{CS}^{i_{3}})$: This query is an eavesdropping attack that the adversary can obtain messages transmitted via a public channel. Thus, this query can be defined as a passive attack.
- $CoUD(U_{US}^{i_{1}})$: In this query, the adversary extracts secret parameters using the smart device of $U_{US}^{i_{1}}$. Therefore, we can define the query CoUD is an active attack.
- $Sn(U_{p}^{i})$: The adversary sends messages to legal participants through open channels. This query is an active attack.
- $Ts(U_{p}^{i})$: In this query, the adversary flips an unbiased coin. When the result of the flipped coin is 0, the session key is not fresh. When the result of the flipped coin is 1, we can demonstrate that the session key is fresh. Otherwise, the result outputs NULL (⊥).

**Theorem** **1.** *We take a definition of* $P_{AD}$, HA, $q_{HA}$, and $q_{Sn}$ *as the possibility of breaking session key, range space of hash function, number of hash functions, and number of send queries, respectively. Moreover, we define that s and C are the Zipf’s parameters [35]. From that, the adversary tries to reveal the session key of the proposed protocol in polynomial time. Following [36,37,38], the ROR model analysis of the proposed protocol is composed of four games (*$GAME_{m}$, m=0,1,2,3*) and the winning possibility of the adversary is* $PW_{GAME_{m}}$ *for each game* $GAME_{m}$*.*

(1) $$P_{AD}\leq \frac{q_{HA}^{2}}{\left|HA\right|}+2\left\{Cq_{S}^{Sn}\right\}$$

- $GAME_{0}$*: In this game, the adversary has no knowledge about the session key. Thus, the adversary picks a random bit B.*
  (2) $$P_{AD}=\left|2PW_{GAME_{0}}-1\right|$$
- $GAME_{1}$*: The adversary conducts* EX *query to collect the messages transmitted via public channels. Thus, the adversary obtains* $\left\{PID_{i},B_{1},B_{2},V_{1},TS_{1}\right\}$*,* $\left\{M_{1},B_{3},V_{2}\right\}$*,* $\left\{B_{6},B_{7},B_{8},V_{3},V_{4},TS_{3}\right\}$*, and* $\left\{B_{8},V_{5},TS_{4}\right\}$*. After that, the adversary flips an unbiased coin to execute the* Ts *query. However, the adversary has no knowledge of the session key* $SK=h(C_{1}\|r_{i}\|r_{j}\|r_{k})$ *because it is composed of random numbers* $r_{i}$*,* $r_{j}$ *and* $r_{k}$ *and masked in the hash functions. For these reasons, the adversary can obtain the following:*
  (3) $$PW_{GAME_{1}}=PW_{GAME_{1}}$$
- $GAME_{2}$*: The adversary conducts* HA *and* Sn *queries to reveal session key in this game. However, the session key is composed of fresh random numbers and a cryptographic hash function. Therefore, the adversary cannot make hash collisions to calculate the session key. Applying the birthday paradox [39], we obtain the following:*
  (4) $$|PW_{GAME_{2}}-PW_{GAME_{1}}|\leq \frac{q_{HA}^{2}}{\left|HA\right|}$$
- $GAME_{3}$*: In the last game, the adversary conducts* CoUD *query to obtain the secret parameters* $\left\{PID_{i},ID_{CS},RPW_{i},A_{1},A_{2},Gen\left(.\right),Rep\left(.\right),\tau_{i}\right\}$*. However, the adversary cannot decrypt the secret parameters because these parameters are encrypted using the identity* $ID_{i}$*, password* $PW_{i}$*, and biometrics* $B_{i}$*. Since simultaneously guessing* $ID_{i}$*,* $PW_{i}$*, and* $B_{i}$ *is a computationally infeasible task, the adversary has no advantage in this game. We obtain the following using Zipf’s law [35].*
  (5) $$|PW_{GAME_{3}}-PW_{GAME_{2}}|\leq Cq_{S}^{Sn}$$
  *When all the games end, the adversary becomes a random bit B.*
  (6) $$PW_{GAME_{3}}=\frac{1}{2}$$
  *We can calculate (7) utilizing (2) and (3).*
  (7) $$\frac{1}{2}P_{AD}=|PW_{GAME_{0}}-\frac{1}{2}|=|PW_{GAME_{3}}-\frac{1}{2}|$$
  *Then, we use (6) and (7) to obtain (8).*
  (8) $$\frac{1}{2}P_{AD}=\left|PW_{GAME_{1}}-PW_{GAME_{3}}\right|$$
  *We calculate (9) utilizing the triangular inequality.*
  $$\frac{1}{2}P_{AD}=\left|PW_{GAME_{1}}-PW_{GAME_{3}}\right|\;\leq |PW_{GAME_{1}}-PW_{GAME_{2}}|\;+|PW_{GAME_{2}}-PW_{GAME_{3}}|$$
  (9) $$\leq \frac{q_{HA}^{2}}{2|HA|}+Cq_{S}^{Sn}$$
  *We calculate (10) multiplying (9) by 2.*
  (10) $$P_{AD}\leq \frac{q_{HA}^{2}}{\left|HA\right|}+2\left\{Cq_{S}^{Sn}\right\}$$
  *We obtain the in Equation (10) which is the same as (1). It means that the adversary cannot distinguish random nonce and the session key using various security attacks, such as* EX*,* CoUD*, and* Sn*. Thus, we can prove the session key security of the proposed protocol.*

### 7.2. BAN Logic

We analyze the mutual authentication of the proposed protocol using BAN logic [40]. Following [41,42,43], we define basic notations and descriptions of BAN logic in Table 2.

#### 7.2.1. Rules

In BAN logic, there are five rules, such as “Message meaning rule (MMR)”, “Nonce verification rule (NVR)”, “Jurisdiction rule (JR)”, “Belief rule (BR)”, and “Freshness rule (FR)”.

**1.**  Message meaning rule (MMR):
  $$\frac{A_{i}\;|\;\equiv \;A_{i}\overset{SH}{↔}A_{j},\;A_{i}⊲{\left\{T_{1}\right\}}_{SH}}{A_{i}\left|\;\equiv \;A_{j}\right|∼T_{1}}$$
**2.**  Nonce verification rule (NVR):
  $$\frac{A_{i}\left|\;\equiv \;\#\left(T_{1}\right),\;A_{i}\right|\equiv A_{j}\;|∼T_{1}}{A_{i}\left|\;\equiv \;A_{j}\right|\equiv T_{1}}$$
**3.**  Jurisdiction rule (JR):
  $$\frac{A_{i}|\;\equiv \;A_{j}⤇T_{1},\;A_{i}\left|\;\equiv \;A_{j}\right|\equiv T_{1}}{A_{i}\;|\;\equiv \;T_{1}}$$
**4.**  Belief rule (BR):
  $$\frac{A_{i}\;|\;\equiv \;\left(T_{1},T_{2}\right)}{A_{i}\;|\;\equiv \;T_{1}}$$
**5.**  Freshness rule (FR):
  $$\frac{A_{i}\;|\;\equiv \;\#\left(T_{1}\right)}{A_{i}\;|\;\equiv \;\#\left(T_{1},T_{2}\right)}$$

#### 7.2.2. Goals

In our protocol, each participant authenticate the communication partner by establishing session key SK. Thus, goals of the proposed protocol can be shown as follows:

**Goal 1:**  $UI|\;\equiv \;UI\overset{SK}{↔}CS$
**Goal 2:**  $UI|\;\equiv \;CS|\;\equiv \;UI\overset{SK}{↔}CS$
**Goal 3:**  $CS|\;\equiv \;UI\overset{SK}{↔}CS$
**Goal 4:**  $CS|\;\equiv \;UI|\;\equiv \;CS\overset{SK}{↔}UI$
**Goal 5:**  $CS|\;\equiv \;CS\overset{SK}{↔}SJ$
**Goal 6:**  $CS|\;\equiv \;SJ|\;\equiv \;CS\overset{SK}{↔}SJ$
**Goal 7:**  $SJ|\;\equiv \;CS\overset{SK}{↔}SJ$
**Goal 8:**  $SJ|\;\equiv \;CS|\;\equiv \;SJ\overset{SK}{↔}CS$

#### 7.2.3. Idealized Forms

In the proposed authentication phase, four messages are transmitted via open channels ($\left\{PID_{i},B_{1},B_{2},V_{1},TS_{1}\right\}$, $\left\{M_{1},B_{3},V_{2}\right\}$, $\left\{B_{6},B_{7},B_{8},V_{3},V_{4},TS_{3}\right\}$, $\left\{B_{8},V_{5},TS_{4}\right\}$). To analyze these messages, we convert them into idealized forms.

$MSG_{1}:UI\to SJ:{\left\{r_{i},ID_{j},TS_{1}\right\}}_{k_{i}}$

$MSG_{2}:SJ\to CS:\{{\left\{r_{i},ID_{j}\right\}}_{k_{i}},{\left\{r_{j},TS_{2}\right\}}_{k_{j}}\}$

$MSG_{3}:CS\to SJ:\{{\left\{r_{k},r_{i},C_{1},TS_{3}\right\}}_{k_{j}},{\left\{r_{j},r_{k},TS_{3}\right\}}_{r_{i}}\}$

$MSG_{4}:SJ\to UI:{\left\{r_{j},r_{k},TS_{4}\right\}}_{r_{i}}$

#### 7.2.4. Assumptions

In the proposed protocol, participants agree on the freshness of the random number and secret parameters. Therefore, we show the assumptions to analyze the proposed authentication phase.

$S_{1}$:
$CS|\;\equiv \;\#(TS_{2})$

$S_{2}$:
$SJ|\;\equiv \;\#(TS_{3})$

$S_{3}$:
$UI|\;\equiv \;\#(TS_{4})$

$S_{4}$:
$CS|\;\equiv \;\#(r_{i})$

$S_{5}$:
$CS|\;\equiv \;UI\overset{k_{i}}{↔}CS$

$S_{6}$:
$CS|\;\equiv \;SJ\overset{k_{j}}{↔}CS$

$S_{7}$:
$SJ|\;\equiv \;CS\overset{k_{j}}{↔}SJ$

$S_{8}$:
$UI|\;\equiv \;CS\overset{r_{j}}{↔}UI$

#### 7.2.5. BAN Logic Proof

**Step 1:**  We obtain $P_{1}$ using $MSG_{2}$.
  $$P_{1}:CS⊲\{{\left\{r_{i},ID_{j}\right\}}_{k_{i}},{\left\{r_{j},TS_{2}\right\}}_{k_{j}}\}$$
**Step 2:**  We use $S_{5}$, $S_{6}$, and MMR to obtain $P_{2}$ and $P_{3}$ from $P_{1}$.
  $$P_{2}:CS|\equiv UI|∼(r_{i},ID_{j})\;P_{3}:CS|\equiv SJ|∼(r_{j},TS_{2})$$
**Step 3:**  From $P_{2}$ and $P_{3}$, we use $S_{1}$, $S_{4}$, and FR to obtain $P_{4}$ and $P_{5}$.
  $$P_{4}:CS|\equiv \#(r_{i},ID_{j})\;P_{5}:CS|\equiv \#(r_{j},TS_{2})$$
**Step 4:**  From $P_{2}$, $P_{3}$, $P_{4}$ and $P_{5}$, we use NVR to obtain $P_{6}$ and $P_{7}$.
  $$P_{6}:CS|\equiv UI|\equiv (r_{i},ID_{j})\;P_{7}:CS|\equiv SJ|\equiv (r_{j},TS_{2})$$
**Step 5:**  We obtain $P_{8}$ using $MSG_{3}$.
  $$P_{8}:SJ⊲\{{\left\{r_{k},r_{i},C_{1},TS_{3}\right\}}_{k_{j}},{\left\{r_{j},r_{k},TS_{3}\right\}}_{r_{i}}\}$$
**Step 6:**  We use $S_{7}$ and MMR to obtain $P_{9}$ from $P_{8}$.
  $$P_{9}:SJ|\equiv CS|∼(r_{k},r_{i},C_{1},TS_{3})$$
**Step 7:**  From $P_{9}$, we use $S_{2}$ and FR to obtain $P_{10}$.
  $$P_{10}:SJ|\equiv \#(r_{k},r_{i},C_{1},TS_{3})$$
**Step 8:**  From $P_{9}$ and $P_{10}$, we use NVR to obtain $P_{11}$.
  $$P_{11}:SJ|\equiv CS|\equiv (r_{k},r_{i},C_{1},TS_{3})$$
**Step 9:**  Using $P_{7}$ and $P_{11}$, CS and SJ computes the session key $SK=h(C_{1}\|r_{i}\|r_{j}\|r_{k})$.
  Thus, we obtain the following:
  $$P_{12}:SJ|\equiv CS|\equiv SJ\overset{SK}{↔}CS\;(\mathrm{Goal}\;8)\;P_{13}:CS|\equiv SJ|\equiv CS\overset{SK}{↔}SJ\;(\mathrm{Goal}\;6)$$
**Step 10:**  Using JR into $P_{12}$ and $P_{13}$, We obtain the following goals:
  $$P_{14}:SJ|\equiv SJ\overset{SK}{↔}CS\;(\mathrm{Goal}\;7)\;P_{15}:CS|\equiv CS\overset{SK}{↔}SJ\;(\mathrm{Goal}\;5)$$
**Step 11:**  We obtain $P_{16}$ using $MSG_{4}$.
  $$P_{16}:UI⊲{\left\{r_{j},r_{k},TS_{4}\right\}}_{r_{i}}$$
**Step 12:**  We use $S_{8}$ and MMR to obtain $P_{17}$ from $P_{16}$.
  $$P_{17}:UI|\equiv CS|∼(r_{j},r_{k},TS_{4})$$
**Step 13:**  From $P_{17}$, we use $S_{3}$ and FR to obtain $P_{18}$.
  $$P_{18}:UI\#(r_{j},r_{k},TS_{4})$$
**Step 14:**  From $P_{17}$ and $P_{18}$, we use NVR to obtain $P_{19}$.
  $$P_{19}:UI|\equiv CS|\equiv (r_{j},r_{k},TS_{4})$$
**Step 15:**  Using $P_{6}$ and $P_{19}$, UI and SJ agrees the session key $SK=h(C_{1}\|r_{i}\|r_{j}\|r_{k})$.
  Thus, we obtain the following:
  $$P_{20}:UI|\equiv CS|\equiv UI\overset{SK}{↔}CS\;(\mathrm{Goal}\;2)\;P_{21}:CS|\equiv UI|\equiv CS\overset{SK}{↔}UI\;(\mathrm{Goal}\;4)$$
**Step 16:**  Using JR into $P_{20}$ and $P_{21}$, We obtain the following goals:
  $$P_{22}:UI|\equiv CS\overset{SK}{↔}UI\;(\mathrm{Goal}\;1)\;P_{23}:CS|\equiv UI\overset{SK}{↔}CS\;(\mathrm{Goal}\;3)$$

### 7.3. Informal Security Analysis

#### 7.3.1. Insider Attack

Malicious actor A, who has gone through the registration phase as a legitimate user, can attempt an insider attack using the acquired information. However, the attacker is unable to know the random values (ri,rj,rk) and $k_{j}$. As a result, the attacker cannot calculate $C_{1}$, rendering the attack impossible.

#### 7.3.2. Impersonation Attack

(1) User impersonation: Adversary *A* needs to create a valid message $M_{1}=\{PID_{i},B_{1},B_{2},$$V_{1},TS_{1}\}$ to impersonate the legitimate user $U_{i}$. While *A* might obtain $PID_{i}$ from the user’s device, it is impossible for *A* to access the necessary $k_{i}$ and $SID_{i}$ to calculate $B_{1},B_{2},V_{1},TS_{1}$ needed to create the message. Therefore, *A* cannot generate the $M_{1}$ message on behalf of the user $U_{i}$ and transmit it to the cloud server and control server. Thus, the proposed scheme is secure against user impersonation attacks.
(2) Cloud server impersonation: To execute this attack, *A* needs to send the message $M_{2}=\left\{M_{1},B_{3},V_{2},TS_{2}\right\}$ to the control server on behalf of the cloud server $S_{j}$. Even if *A* intercepts the transmission of $M_{1}$ over an open channel, they cannot generate the necessary $B_{3},V_{2}$ for the message until they know $k_{j}$. Therefore, the proposed scheme is secure against cloud server impersonation attacks.

#### 7.3.3. Reply and MITM Attacks

All users, the cloud server, and the control server attempt to validate the received messages through $V_{1}^{\prime },V_{2}^{\prime },V_{3}^{\prime },V_{4}^{\prime }$. Also, the sent messages are masked with different random values for each session, ensuring freshness. Therefore, the proposed scheme is secure against reply attacks and MITM attacks.

#### 7.3.4. Privileged Insider Attack

In this attack scenario, external entity A is considered a privileged insider, implying that A possesses the user’s registration request message $ID_{i}$ and confidential values such as $\left\{A_{1},A_{2},ID_{CS},Gen\left(\cdot \right),Rep\left(\cdot \right),\tau_{i}\right\}$ However, without the precise biometric information, ID, or PW values of the user, calculating $k_{i}=A_{1}⊕(ID_{i}||PW_{i})$ or $SID_{i}=A_{2}⊕(\sigma_{i}||PW_{i})$ is not possible. As a result, the proposed scheme is secure against privileged insider attacks.

#### 7.3.5. Ephemeral Security Leakage Attack

To prevent adversary *A* from carrying out valid attacks, such as obtaining the session key through this attack scenario, it is essential to ensure that the session key is preserved even if the random values used in the session are exposed. Therefore, assuming *A* knows the values of $r_{i},r_{j},r_{k}$, it is postulated here that even with this knowledge, *A* cannot calculate SK without knowing $SID_{i},SID_{j}$. Additionally, valid attacks like impersonating the user or cloud server using random values are not possible. Therefore, the proposed scheme is secure against ESL attacks.

#### 7.3.6. Stolen Verifier Attack

We can assume that a malicious *A*, upon obtaining $\left\{A_{3}\right\}$ from the cloud server’s database, attempts to calculate the session key $SK=h(C_{1}||r_{i}||r_{j}||r_{k})$ or impersonate the cloud server. However, without the cloud server’s secret key $x_{j}$, *A* cannot deduce the value of $k_{j}$ from the stored $A_{3}$, nor can *A* determine the randomly generated values $r_{i},r_{j}$, or $r_{k}$. Therefore, *A* is unable to compute the session key or impersonate the cloud server. Consequently, the proposed scheme is secure against verification table leakage attacks.

#### 7.3.7. DoS Attack

The adversary *A* may intentionally attempt to send the message $M_{1}=\{PID_{i},B_{1},B_{2},V_{1},$$TS_{1}\}$ repeatedly. However, to generate message $M_{1}$, *A* must go through the login process and pass the verification $RPW_{i}^{\prime }\overset{?}{=}RPW_{i}$. However, to create a valid $RPW_{i}^{\prime }=h(ID_{i}||PW_{i}\left|\right|\sigma_{i})$, *A* cannot have the required $ID_{i},PW_{i},\sigma_{i}$. Therefore, *A* cannot create and repeatedly send the message $M_{1}$, making the proposed scheme secure against DoS attacks.

#### 7.3.8. User Anonymity and Untraceability

Due to the use of $PID_{i}$ as a pseudo identity, the user’s identity $ID_{i}$ cannot be deduced by an adversary *A*. Additionally, the $PID_{i}$ is updated as a new value with random elements for each session, making it impossible for *A* to compare $PID_{i}$ values between previous and current sessions to compromise the user’s untraceability. Therefore, the proposed scheme provides user anonymity and untraceability.

#### 7.3.9. Session Key Disclosure Attack

To calculate the session key $SK=h(C_{1}||r_{i}||r_{j}||r_{k})$, adversary *A* needs to have access to the values of $SID_{i}$, $SID_{j}$, $r_{i}$, $r_{j}$, and $r_{k}$. However, for *A* to discover $SID_{i}$, $SID_{j}$, they would need access to the secret key $x_{cs}$ and the random values $n_{i}$ and $r_{k}$. Additionally, random values like $r_{i}$, $r_{j}$, $r_{k}$ are used temporarily and exist only within a single session. Therefore, the proposed scheme is secure against session key disclosure attacks.

#### 7.3.10. Perfect Forward Secrecy

If the control server’s secret key $x_{cs}$ is compromised, adversary *A* may attempt to calculate the session key SK for a previous session. However, since $SK=h(C_{1}||r_{i}||r_{j}||r_{k})$ does not contain $x_{cs}$ and the values of $r_{i},r_{j},r_{k}$ are random and cannot be deduced, A cannot perform the calculation. Furthermore, without $n_{i}$ through $x_{cs}$, A cannot compute $SID_{i}$. Therefore, the proposed scheme ensures perfect forward secrecy.

#### 7.3.11. Mutual Authentication

In the login and authentication phases, the messages $\left\{PID_{i},B_{1},B_{2},V_{1},TS_{1}\right\}$ and $\left\{M_{1},B_{3},V_{2},TS_{2}\right\}$ included can be used by the control server to verify the legitimacy of the user and the cloud server through the transmitted $V_{1}$ and $V_{2}$. Additionally, messages $\left\{B_{6},B_{7},B_{8},B_{9},V_{3},V_{4},TS_{3}\right\}$ and $\left\{B_{8},B_{9},V_{4},TS_{4}\right\}$ allow both the user and the cloud server to validate each other’s identity using $V_{3}$ and $V_{4}$. Due to the unavailability of $SID_{i}$, kj values, and random values to adversaries through the open channel, the transparency of authentication is ensured. Therefore, the provided scheme offers mutual authentication.

## 8. Performance Analysis

### 8.1. Security Features Comparison

We visually compare the safety elements of the proposed scheme and related schemes [11,21,44,45,46,47,48] and record them in Table 3, which includes various types of safety elements such as “insider attack”, “impersonation attack”, “stolen verification attack”, “ESL attack”, “privileged attack”, “perfect forward secrecy”, “reply attack”, “offline password-guessing attack”, “session key disclosure”, “mutual authentication”, “DoS attack”, “user anonymity”, and “untraceability”. Ultimately, the proposed scheme offers more security features compared to Wu et al.’s scheme, and it exhibits fewer features that are either unidentified or not provided, even when compared to the schemes of other related works.

### 8.2. Communication Costs Comparison

**Figure 8:**
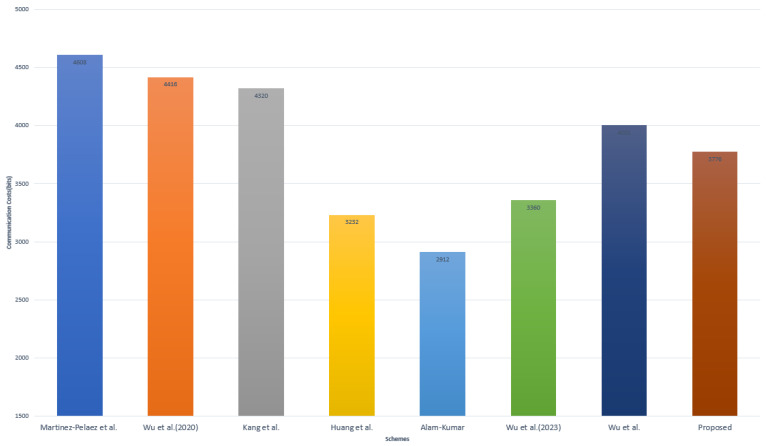
Communication costs comparison [7,11,17,21,40,41,42,43,44,45,46,47,48].

We conducted a comparative analysis of communication costs between the related schemes [11,21,44,45,46,47,48] and the proposed scheme. Based on [11], we assume the bit lengths of hash function, timestamp, string, identity, random number, fuzzy extractor, and encryption operation to be 256, 32, 160, 160, 160, 8, and 256 bits, respectively. Therefore, during the MAKA phase of our proposed scheme, the exchanged message $M_{1}=\left\{PID_{i},B_{1},B_{2},V_{1},TS_{1}\right\}$ requires (160 + 160 + 160 + 256 + 32 = 768 bits), message $M_{2}=\left\{M_{1},B_{3},V_{2},TS_{2}\right\}$ requires (768 + 160 + 256 + 32 = 1216 bits), message $M_{3}=\{B_{6},B_{7},B_{8},B_{9},V_{3},$$V_{4},TS_{3}\}$ requires (160 + 160 + 160 + 160 + 256 + 256 + 32 = 1184 bits), and message $M_{4}=\left\{B_{8},V_{5},TS_{4}\right\}$ requires (160 + 160 + 256 + 32 = 608 bits). Table 4 and Figure 8 present a summary of the communication costs for the associated schemes [11,21,44,45,46,47,48] and the proposed scheme.

### 8.3. Computation Costs Comparison

**Figure 9:**
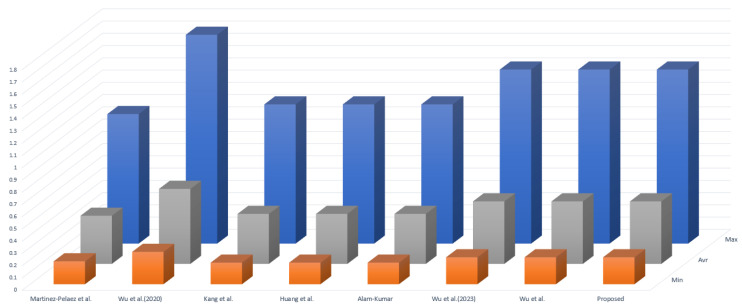
Computation costs comparison on user side devices [7,11,17,21,40,41,42,43,44,45,46,47,48].

We conducted a comparative analysis of computation costs for the AKA phase of the proposed scheme and related schemes [11,21,44,45,46,47,48]. Based on [49], we designed the environment for computing costs. The experimental environment and the performance of operation costs, including the minimum, maximum, and average values, are summarized in Table 5. We represent hash function as $T_{h}$ and encryption/decryption operations of AES-256 as $T_{e}$. Using these values, we conducted a comparison of computation costs as shown in Table 6 and Figure 9.

We can observe that the computational costs for users using the proposed scheme and users using Wu et al.’s scheme are the same. Next, we calculated the computational costs of the cloud server and control server for the proposed scheme and related schemes based on the environments provided in [49] as well. Table 7 represents the calculated computational costs for the proposed scheme and related schemes.

When comprehensively examining the results of the comparison with related schemes, we can elaborate as follows. Our proposed scheme offers more security elements compared to other schemes and is secure against various attacks such as insider attacks, impersonation attacks, stolen verification attacks, ESL attacks, privileged insider attacks, reply attacks, and offline password-guessing attacks. Simultaneously, it maintains reasonable user-side computation cost and communication cost suitable for the CloudIoT environment. However, it is noteworthy that to provide such robust security, additional computation operations on the server side have been introduced.

## 9. Conclusions

This study analyzed the key agreement protocol between cloud-enabled IoT devices and cloud servers as proposed by Wu et al. The scheme proposed by Wu et al. was found to be vulnerable to insider, privileged insiders, impersonation, and verification table leakage attacks and lacks user untraceability. In addition, it is inconvenient for users to update their passwords offline. To overcome these vulnerabilities and inconveniences, this study proposed a provably secure lightweight MAKA protocol for the cloud-based IoT environments.

The proposed protocol ensures safety against various attacks by preventing the exposure of critical parameters using user biometric information, and the cloud server’s secret key. Furthermore, user untraceability was ensured by updating the user’s pseudonym in every session and convenience was enhanced by adding an offline user password change and biometric template update phase. The safety of mutual authentication and the resulting session key was verified using the RoR model and BAN logic. Moreover, informal analysis was conducted to verify safety against attacks such as insider attacks, impersonation attacks, privileged attacks, ESL attacks, stolen verifier attacks and DoS attacks, while confirming security features such as user anonymity, untraceability, and perfect forward secrecy. The security features, communication costs, and computation costs of the proposed scheme were compared. This comparison demonstrated that the proposed scheme is rational in terms of communication and computation amounts in the cloud-based IoT environments, while being verified for safety.

In conclusion, the proposed scheme demonstrated robust safety and the ability to provide users with real-time services securely. Future research will focus on integrating the proposed scheme into real-world environments and various industrial settings where cloud-based IoT is applied.

## Figures and Tables

**Table 1:** Authentication scheme overview.

Schemes	Cryptographic Technologies	Limitaion
Islam-Biswas [23]	- ECC - Self-certified public keys	- Cannot provide anonymity - Vulnerable to reply and clogging attacks
He et al. [28]	- Asymmetric cryptography	- Vulnerable to insider attacks, offline password-guessing, user impersonation attacks, and DoS attacks
Chen at al. [25]	- XOR operation - Hash function - Elliptic multiplication	- Vulnerable to stolen smartcard, offline password-guessing, offline identity guessing, and reply attacks
Prosanta-Biplab [18]	- PUF - Fuzzy extractor	-Vulnerable to man-in-the-middle attacks, impersonation attacks, session key hijaking, conventional and differential template attacks
Zhou et al. [20]	- XOR operation - Hash function	- Cannot provide mutual authentication - Vulnerable to insider attacks and man-in-the-middle attacks
Nikooghadam et al. [13]	- XOR operation - Hash function	- Vulnerable to reply attacks, privileged insider attacks, offline password-guessing, known key temporary imformation attacks, server spoofing attacks and impersonation attcks
Tsai-Lo [29]	- Single Sign On scheme	- Cannot provide sesssion key security, mutual authentication, and user anonymity - Vulnerable to impersonation attacks
Kumari et al. [30]	- Hash function - Diffie-Hellman	- Cannot provide user unlinkability and anonymity, data confidentiality - Vulnerable to known session-specific temporary information attacks, impersonation attacks, and desynchronization attacks
Bhuarya et al. [31]	- ECC	- Cannot provide mutual authentication - Vulnerable to impersonation attacks and man-in-the-middle attacks

**Table 2:** Basic notations and decriptions.

Notation	Description
$A_{i},A_{j}$	Principals
SK	Session key
$T_{1},T_{2}$	Statements
$A_{i}|\;\equiv \;T_{1}$	$A_{i}$ **believes** $T_{1}$
$A_{1}|\;∼\;T_{1}$	$A_{i}$ once **said** $T_{1}$
$A_{i}\;⤇\;T_{1}$	$A_{i}$ **controls** $T_{1}$
$A_{i}\;⊲\;T_{1}$	$A_{i}$ **receives** $T_{1}$
$\#T_{1}$	$T_{1}$ is **fresh**
${\left\{T_{1}\right\}}_{S}$	$T_{1}$ is **encrypted** with *S*
$A_{i}\overset{SH}{↔}A_{j}$	$A_{i}$ and $A_{j}$ have a shared key SH

**Table 3:** Security and functionality features(SFF) comparison.

SFF	[21]	[44]	[45]	[46]	[47]	[48]	[11]	Proposed
*SP1*	✓	✓	✓	✓	Δ	Δ	×	✓
*SP2*	×	✓	✓	×	✓	✓	×	✓
*SP3*	✓	✓	Δ	✓	Δ	Δ	×	✓
*SP4*	✓	✓	Δ	×	✓	✓	✓	✓
*SP5*	✓	✓	Δ	×	✓	✓	×	✓
*SP6*	✓	✓	Δ	✓	✓	Δ	✓	✓
*SP7*	×	✓	✓	✓	Δ	✓	✓	✓
*SP8*	✓	✓	×	✓	✓	Δ	✓	✓
*SP9*	×	✓	✓	×	Δ	Δ	×	✓
*SP10*	×	✓	✓	✓	Δ	✓	✓	✓
*SP11*	✓	✓	Δ	✓	Δ	Δ	✓	✓
*SP12*	×	✓	✓	✓	✓	✓	✓	✓
*SP13*	✓	✓	Δ	✓	✓	✓	×	✓

Note: SP1: insider attack; SP2: impersonation attack; SP3: stolen verification attack; SP4: ESL attack; SP5: privileged insider attack; SP6: perfect forward secrecy; SP7: reply attack; SP8 offline password-guessing attack SP9: session key disclosure; SP10: mutual authentication; SP11: DoS attack; SP12: user anonymity; SP13: untraceability; ✓: provides safety/functional features; ×: does not provides safety/functional features; Δ: not verified.

**Table 4:** Comparison analysis of communication costs.

Scheme	Total Cost (bits)	Number of Messages
Martinez-Pelaez et al. [21]	4608 bits	6
Wu et al. (2020) [44]	4416 bits	5
Kang et al. [45]	4320 bits	4
Huang et al. [46]	3232 bits	4
Alam-Kumar [47]	2912 bits	4
Wu et al. (2023) [48]	3360 bits	4
Wu el al. [11]	4001 bits	5
Proposed	3776 bits	4

**Table 5:** Hardware software enviroment and operation costs.

Hardware/Software	Operation	Max	Min	Average
Raspberry PI 4B with Linux Ubuntu 18.04.4 LTS with 64-bits, 8 GB, and MIRACL library	Hash function $T_{h}$	0.142 ms	0.022 ms	0.051 ms
	AES-256 $T_{e}$	0.021 ms	0.011 ms	0.012 ms

**Table 6:** Comparison analysis of user side computation costs.

Protocol	User Side	Max	Min	Average
[21]	$7T_{h}+3T_{e}$	≈1.057 ms	≈0.187 ms	≈0.393 ms
[44]	$12T_{h}$	≈1.704 ms	≈0.264 ms	≈0.612 ms
[45]	$8T_{h}$	≈1.136 ms	≈0.176 ms	≈0.408 ms
[46]	$8T_{h}$	≈1.136 ms	≈0.176 ms	≈0.408 ms
[47]	$8T_{h}$	≈1.136 ms	≈0.176 ms	≈0.408 ms
[48]	$10T_{h}$	≈1.42 ms	≈0.22 ms	≈0.51 ms
[11]	$10T_{h}$	≈1.42 ms	≈0.22 ms	≈0.51 ms
Proposed	$10T_{h}$	≈1.42 ms	≈0.22 ms	≈0.51 ms

**Table 7:** Comparison analysis of cloud server side control server side computation costs.

Scheme	Cloud Server	Control Server	Total Average (ms)
[21]	$5T_{h}+3T_{e}$	$21T_{h}+2T_{e}$	≈1.386 ms
[44]	$8T_{h}$	$19T_{h}$	≈1.377 ms
[45]	$4T_{h}$	$11T_{h}$	≈0.765 ms
[46]	$4T_{h}$	$10T_{h}$	≈0.714 ms
[47]	$3T_{h}$	$6T_{h}$	≈0.459 ms
[48]	$5T_{h}$	$12T_{h}$	≈0.867 ms
[11]	$5T_{h}$	$13T_{h}$	≈0.918 ms
Proposed	$6T_{h}$	$16T_{h}$	≈1.122 ms

## Data Availability

Data are contained within the article.
